# The Effects of Electronic Cigarette (ECIG)-Generated Aerosol and Conventional Cigarette Smoke on the Mucociliary Transport Velocity (MTV) Using the Bullfrog (*R. catesbiana)* Palate Paradigm

**DOI:** 10.3389/fphys.2017.01023

**Published:** 2017-12-11

**Authors:** Dominic L. Palazzolo, John M. Nelson, Emily A. Ely, Andrew P. Crow, James Distin, Stan C. Kunigelis

**Affiliations:** Department of Physiology, DeBusk College of Osteopathic Medicine, Lincoln Memorial University, Harrogate, TN, United States

**Keywords:** ECIG, E-liquid, vaping, smoking, aerosol, frog palate, MTV, SEM

## Abstract

**Background:** While ECIGs are under scrutiny concerning safety, particularly in reference to the physiological impact that aerosolized ECIG liquid (E-liquid) may have on respiratory tissues, others believe that ECIGs are a “Harm Reduction” alternative to conventional cigarettes. Previous studies investigating ciliated respiratory epithelium indicate that smoking shortens cilia length, reduces cilia beat frequency and disrupts respiratory epithelium, which most likely contributes to the inhibition of mucocilliary clearance. Monitoring mucous clearance of respiratory tissues exposed to ECIG-generated aerosol or conventional cigarette smoke, as indexed by mucous transport velocity (MTV), is one way to gauge the impact aerosol and smoke have on the respiratory tract. Therefore, we designed an experiment to test the effect of ECIG-generated aerosol and smoke on MTV using the frog palate paradigm.

**Methods:** Peristaltic pumps transport ECIG-generated aerosol and conventional cigarette smoke into custom-made chambers containing excised bullfrog palates. MTVs were determined before exposure, immediately after exposure and approximately 1 day following exposure. MTVs were also determined (at the same time points) for palates exposed to air (control). Surface and cross sectional SEM images of palates from all three groups were obtained to support MTV data.

**Results:** The results indicate that ECIG-generated aerosol has a modest inhibitory effect (*p* < 0.05) on MTV 1 day post-exposure (0.09 ± 0.01) compared to control MTV (0.16 ± 0.03 mm/s). In contrast, smoke completely inhibits MTV from 0.14 ± 0.03 mm/s immediately before exposure to 0.00 mm/sec immediately after exposure and the MTV is unable to recover 1 day later. SEM images of control palates and palates exposed to ECIG-generated aerosol both show cilia throughout their epithelial surface, while some areas of palates exposed to smoke are completely devoid of cilia. Additionally, the epithelial thickness of aerosol-exposed palates appears thicker than control palates while smoke-exposed palates appear to be thinner due to epithelial disruption.

**Conclusions:** These results indicate that ECIG-generated aerosol has only a modest effect on mucocilary clearance of bullfrog palates and aerosol sedimentation accounts for epithelial thickening. In accordance with the primary literature, conventional cigarette smoke dramatically inhibits mucociliary clearance and is, in part, due to decreased number of cilia and disruption of the smoke-exposed epithelium.

## Introduction

From their introduction in China, in 2003, ECIGs have quickly become extremely popular and pervasive worldwide. The use of ECIGs is currently under considerable scrutiny by those who believe there is not enough information concerning the physiological impact that the composition of aerosolized ECIG liquid (E-liquid) may have on human health (Palazzolo, 2013). Two recent and highly publicized papers report the presence of formaldehyde in ECIG-generated aerosols (Jensen et al., 2015) and DNA strand breaks and cell death induced by ECIG vapor (Yu et al., 2016). These reports claim that vaping is as or more dangerous than traditional smoking. On the other hand, others believe that ECIGs can be used effectively as a “Harm Reduction” alternative to conventional cigarettes since the detrimental constituents and ingredients that make up E-liquid (and, by extension, to ECIG-generated aerosol) are minimally toxic (Levy et al., 2017). Furthermore, since tobacco is not burned, the thousands of toxic compounds associated with the combustion of tobacco are not inhaled (Talhout et al., 2011). Even the new “heat-not-burn” tobacco products (iQOS), touted by “Big Tobacco” as a safer alternative to conventional cigarettes, are known to emit carcinogenic aldehyde compounds, such as formaldehyde, acetaldehyde and acrolein at higher concentrations emitted by ECIG devices, although substantially lower than what is emitted by conventional cigarettes (Ruprecht et al., 2017). Consequently, the debate over ECIG safety vs. “Harm Reduction” continues (Bhatnagar et al., 2014; Chapman, 2014; Oh and Kacker, 2014; Pisinger, 2014; Abrams and Niaura, 2015). Regardless of whether the use of ECIG by ex-smokers presents hidden perils or a lifesaving haven, there is still much that is not known about the effects and risks of ECIG use, particularly when it comes to inhalation of aerosol.

Because ECIG-generated aerosol, like conventional cigarette smoke, is inhaled directly into the oral cavity, the mucosal surface of the respiratory tract is the first tissue to receive the assault. In humans, it is known that cigarette smoke shortens cilia length (Hessel et al., 2014) and reduces cilia beat frequency (Agius et al., 1997), which most likely contributes to the inhibition of mucocilliary clearance within the large and small airways of the respiratory system (Lourenco et al., 1971; Hessel et al., 2014). The frog palate paradigm is a well-established model commonly used to study mucociliary clearance (Zayas et al., 2004). By using this paradigm, we established a system to assess *ex-vivo* mucous transport velocity (MTV), an index of mucocilliary clearance, of palates exposed to ECIG-generated aerosol and conventional cigarette smoke. Histological observation of palates using scanning electron microscopy (SEM) supplement the MTV data. Furthermore, these results provide valuable insight into the potential effects ECIG-generated aerosol may have on the respiratory tract of humans.

## Methods

### Chemicals

Tricaine methane sulfonate (MS-222) used for frog euthanasia, charcoal powder for determination of MTV and all chemicals used to make Frog Ringer Solution (0.8 gm NaCl, 0.02 gm KCl, 0.02 gm CaCl_2_ anhydrous, 0.02 gm NaHCO_3_, per 100 mL of distilled water, adjusted to pH 7.4 and supplemented with 300 units/mL of penicillin and 300 μg/mL streptomycin) were purchased through Thermo-Fischer Scientific (Waltham, MA).

### Animals and housing

Large adult bullfrogs (≈5–6 inches and 400–500 grams each), without regard to sex or seasonal conditions, were purchased from Charles D. Sullivan Co. (Amphibians of North America, Nashville, TN). All frogs were acclimated to the university animal housing facility (thermostatically controlled at 21 ± 2°C with a 12 h light/dark cycle) in covered aquariums (10 gallon total volume) containing no more than two to three gallons of conditioned tap water (i.e., pH 5.5–8.5, nitrites ≈ 0 ppm, nitrates <40 ppm as determined by aquarium test strips; chloramines were removed by commercially available water conditioners; and the water was aerated using an ambient air pump) at room temperature for at least 1 day before harvesting of palates for MTV determinations. The bottom of each aquarium contained river rocks to mimic a natural interphase between land and water. Frogs were maintained under these conditions for no more than 2 days before euthanasia. To minimize stress, only three to four frogs were housed in any one aquarium at any given time. All aquariums were thoroughly washed following euthanasia of frogs so that the next batch of frogs could be accommodated. The acquisition and handling of these animals complied with the Collaborative Institutional Training Initiative (CITI) Program for animal care and use specific to amphibians in a research setting. All pertinent certificates of training are currently on file with the Lincoln Memorial University (LMU) Office of Research, Grants and Sponsored Programs. This study was carried out in accordance with the recommendations of the Institutional Animal Care and Use (IACU) Guidebook and approved by the LMU IACU committee (protocol number is 14031701-B).

### Harvesting of frog palates and experimental design

Bullfrogs were euthanized by immersion into tap water containing 5 gm/liter of tricaine methane sulfonate (MS-222) for 60 min (AVMA Guidelines for the Euthanasia of Animals, 2013)^1^ The upper palate of each frog was excised, and cut along the mid sagittal line to yield two half-palates as described by Zayas et al. (2004). Each half-palate was supported by a 2 × 2 square inch of medical gauze soaked with frog ringer solution (FRS) and distilled water in a 2:1 v/v. In turn, the gauze was placed in a 3-inch diameter polyethylene petri dish, which also contained 2:1 FRS. This allowed the gauze to remain moist and keep the half-palates suspended above the FRS while still preventing them from drying out. The petri dishes were covered and placed in the refrigerator (4°C) for up to 24 h and were only removed from the refrigerator during the time it took to determine MTVs. The palates were divided into five treatment groups (external control, internal control for aerosol, aerosol, internal control for smoke and smoke) with 8–10 half-palates *per* group. The half-palates of the external control group are the never exposed control. The aerosol group consisted of half-palates exposed to ECIG-generated aerosol and the corresponding half-palates exposed to air served as its internal control. Similarly, the smoke group consisted of half-palates exposed to smoke from conventional cigarettes and the corresponding half-palates exposed to air served as its internal control. The MTVs for all groups were determined 1, 2 and 24 h post euthanasia. The internal control groups for aerosol and smoke were exposed to 45 puffs of air immediately before the MTV was determined at the 2-h post euthanasia time point. Similarly, the aerosol and smoke groups were exposed to 45 puffs of aerosol or smoke immediately before the MTV was determined at the 2-h post euthanasia time point. This experimental design is outlined in Table 1.

**Table 1:** Time Line for MTV determination.

**Group**	**Post euthanasia exposure times**		
	**1-h**	**2-h**	**24-h**
External Control	Never Exposed (*n* = 8)	Never Exposed (*n* = 8)	Never Exposed (*n* = 8)
Internal Control for Aerosol	Pre-exposure (*n* = 10)	Exposure to Air (*n* = 9)	Post-exposure (*n* = 10)
Aerosol	Pre-exposure (*n* = 10)	Exposure to Aerosol (*n* = 9)	Post-exposure (*n* = 10)
Internal Control for Smoke	Pre-exposure (*n* = 8)	Exposure to Air (*n* = 9)	Post-exposure (*n* = 10)
Smoke	Pre-exposure (*n* = 8)	Exposure to Smoke (*n* = 9)	Post-exposure (*n* = 10)

*n, number of half-palates*.

### Exposure of palates to air, aerosol or smoke

**Figure 1:**
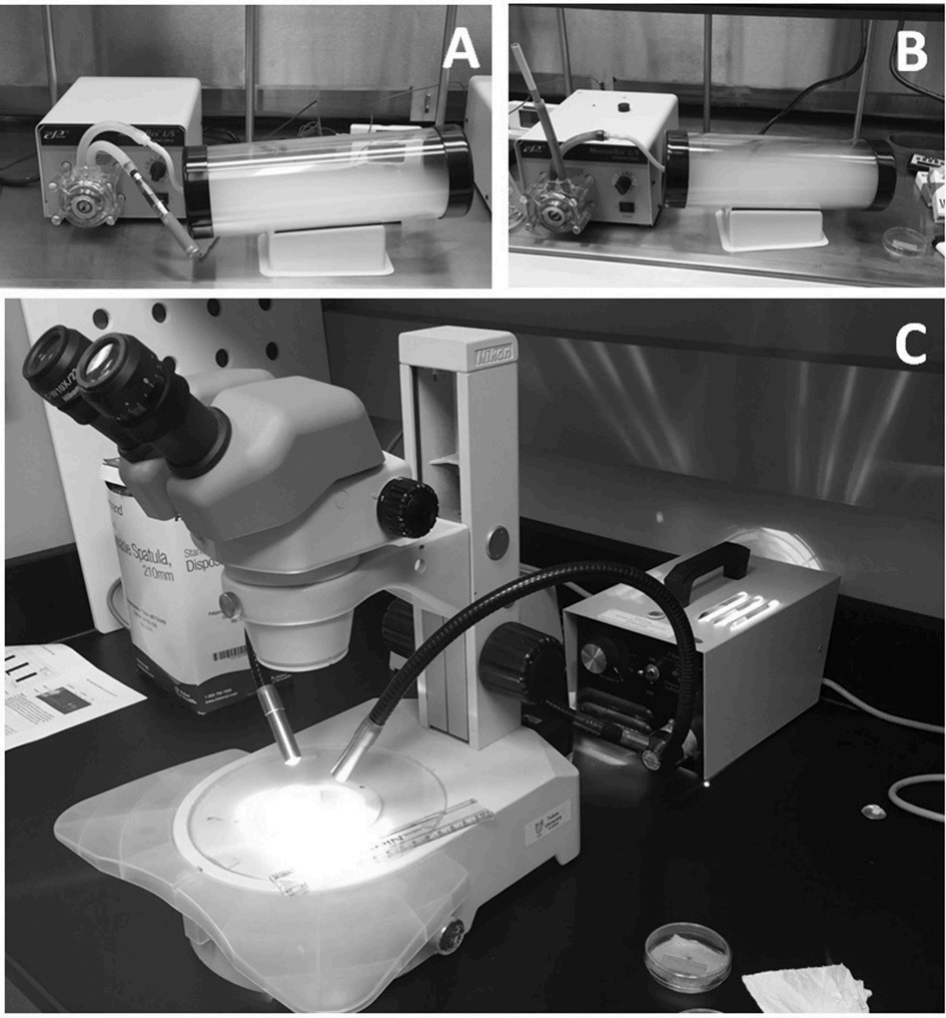
Equipment used for half-palate exposures to **(A)** air and aerosol, and **(B)** air and smoke. MTVs were determined by observing the rate at which charcoal powder moves across the surface of the frog palate using a stereomicroscope **(C)**.

Petri dishes containing the half-palates were placed into clear cylindrical acrylic exposure chambers uncovered and subsequently exposed to air (internal controls), ECIG-generated aerosol or conventional cigarette smoke. The dimensions of the cylinders are 30 cm in length, with an internal diameter of 9.5 cm and a wall thickness of 3 mm (chamber volume is 2,126 cm^3^). Each end of the cylinder is closed off with tight fitting rubber caps. The inlet cap to the chamber had a small hole (4.762 mm diameter) in the center so that the outlet tube from a peristaltic pump can introduce air, aerosol or smoke (Figures 1A,B). The outlet cap of the chamber had a similar small hole in the center to allow air, aerosol or smoke to escape. Air, aerosol or smoke were pumped into the chambers in a setup similar to that previously described (Palazzolo et al., 2017). Briefly, two Cole-Parmer Master Flex L/S peristaltic pumps (Vernon Hills, IL) were used to simulate puffing on Triple 3 (Kennesaw, GA) eGo style ECIG devices or conventional Marlboro® (84 mm, full strength) cigarettes. The Triple 3 eGo devices, manufactured in China by JOMO Tech, consist of a 650 mAh lithium ion battery (3.7 V, unregulated), a silicone ring at the base of the mouth piece, and a plastic tank (i.e., “clearomizer”) with a 1.6 ml capacity to house the E-liquid. The resistance of the tank's heating coils varies between 2.2 and 2.6 Ω for an average power output of ≈5.7 W. The ECIG devices vaporized a commercially available E-liquid (7 s, tobacco flavor, very high nicotine; South Lake, TX) mixture of 80% propylene glycol and 20% vegetable glycerin (i.e., glycerol) containing 24 mg/ml of nicotine or approximately 3.4 mg nicotine/15 puffs. In comparison, a full-strength Marlboro® contains slightly less than 1.0 mg nicotine/cigarette Calafat et al. (2004). One peristaltic pump (aerosol pump) was used to transport air or mainstream ECIG-generated aerosol through ≈40 inches of Master Flex L/S 24 Precision Tubing (ID = 6.4 mm) into the exposure chamber. The outlet tubing from the pump was connected to a four-inch length of Fisherbrand Tygon S3 flexible tubing (*ID* = 3.175 mm, *OD* = 4.762 mm) using a small plastic downsizing connector. This smaller diameter tubing is inserted through the small hole located in the center of the rubber inlet cap so that air or aerosol could be introduced into the chamber. A second peristaltic pump (the smoke pump) was used to transport air or mainstream smoke through an identical setup as the first peristaltic pump. To minimize cross contamination of pump tubing, the aerosol pump was used strictly for aerosol and the smoke pump strictly for smoke. Before each air, aerosol or smoke trial, pump flow rates were equilibrated to 400 ml/min using an Aalborg GFM flow meter (Orangeburg, NY) to simulate the flow of air intake during a 5-s puff on an ECIG device or conventional cigarette. The puffing protocol consisted of 45 cycles of a 5 s puff (pump active) followed by a 10 s rest period (pump inactive), where 15 puffs approximates the extent of one cigarette. The petri dishes containing the half-palates were placed into the exposure chambers for subsequent exposure to air, aerosol, or smoke. Every pump-puffing experiment was conducted within a Thermo Scientific Hamilton SafeAire II (Fisher Hamilton L.L.C., Two Rivers, WI) laminar flow hood (≈0.6 MPS) equipped with a HEPA filter.

### Determination of palate MTV

MTVs were determined 1- (pre-exposure), 2- (exposure), and 24- (post exposure) hours post euthanasia. At the time of MTV assessment, petri dishes were removed from the refrigerator, uncovered and a small quantity of 2:1 FRS was siphoned from the petri dish using a disposable plastic transfer pipette to bathe the surface of the half-palate. Next, a few particles of charcoal powder were gently placed on the surface of the half-palates. The half-palates were placed on the stage of a Nikon SMZ800 Zoom stereomicroscope (Melville, NY) equipped with a calibrated 0–10 mm standard reticle on a 10X eyepiece. The MTV was determined by observing the time, in seconds, the particles of charcoal moved across 10 mm of the palate surface (Figure 1C) and then recorded as a rate (mm/sec). To minimize variability in MTV determination, the amount of time in which palates sat in ambient room temperature before, during and after exposures was kept constant and only particles of charcoal particles <0.2 mm diameter were used.

### SEM analysis of palates

An additional three groups (*n* = 3/group) of palates were harvested for SEM analysis. One group consisted of never exposed palates, the second group consisted of palates exposed to 45 puffs of aerosol 2-h post euthanasia and the third group consisted of palates exposed to 45 puffs of smoke 2-h post euthanasia. Following puff-exposures, all palates from each group were cut into eight pieces of approximately the same size and trimmed for ease of mounting and eventual SEM surface or cross sectional viewing. The palates were fixed for 24-h with 2.5% glutaraldehyde in phosphate buffered saline (PBS). The fixation solution was removed by twice rinsing the palates for 20 min with deionized water. Palates were then placed in 1% osmium tetroxide for 24-h to facilitate lipid fixation and then run through a four-step process of increasing ethanol gradient (25, 50, 70, and 90%) in which each step lasted 1 h. The palates were held overnight in 100% ethanol. The next morning, the palates were chemically dried using a 2:1 ratio of 100% ethanol to hexamethyldisilizane for 1 h followed by a 1:1 ratio of 100% ethanol to hexamethyldisilizane overnight. The following morning, all palates were dried and mounted to 13 mm diameter aluminum pin-type studs (Structure Probe, Inc. (SPI), West Chester, PA) using 12 mm diameter conductive, double-sided, carbon-impregnated adhesive discs (SPI). Palates were secured to the studs to optimize surface (palate surface facing up for viewing of epithelium) or cross sectional (palate crosscut facing up for viewing of epithelial thickness) viewing. The mounted specimens were then placed into a Hummer IV-A Sputtering System (Anatech Ltd., Alexandria, VA) and coated with 300 Å of 1:1 gold/palladium. A LEO 982 electron microscope (Zeiss, Germany) field emission SEM was used to capture the topography of the palate epithelium, the epithelial thickness and the submucosal collagen arrangement. In addition, a Hitachi TM3000 (Hitachi, High-Technologies Corp, Dallas, TX) tabletop SEM equipped with a Bruker Quantax 70 (Bruker Optics, Billerica, MA) energy-dispersive X-ray (EDX) spectrometer was used to surveil the relative percent composition of carbon (C), oxygen (O), and nitrogen (N) atoms on the palate surface. All SEM images using the LEO 982 electron microscope were captured at an acceleration voltage of 5 kV and depicted at 1000X and 5000X for palate surface topography and 220X and 500X for palate cross sections.

### Statistical analysis

Mean ± SE were determined for MTVs and relative amounts of C, O, and N. Statistical variance between MTV groups was determined using a two-way ANOVA, followed by Bonferroni post hoc analysis. One-way ANOVA followed by Tukey *post hoc* analysis was used to determine statistical variance between the relative amounts of C, O, and N, and palate epithelial thickness. Differences were considered statistically significant when *p* < 0.05.

## Results

### MTV analysis

**Figure 2:**
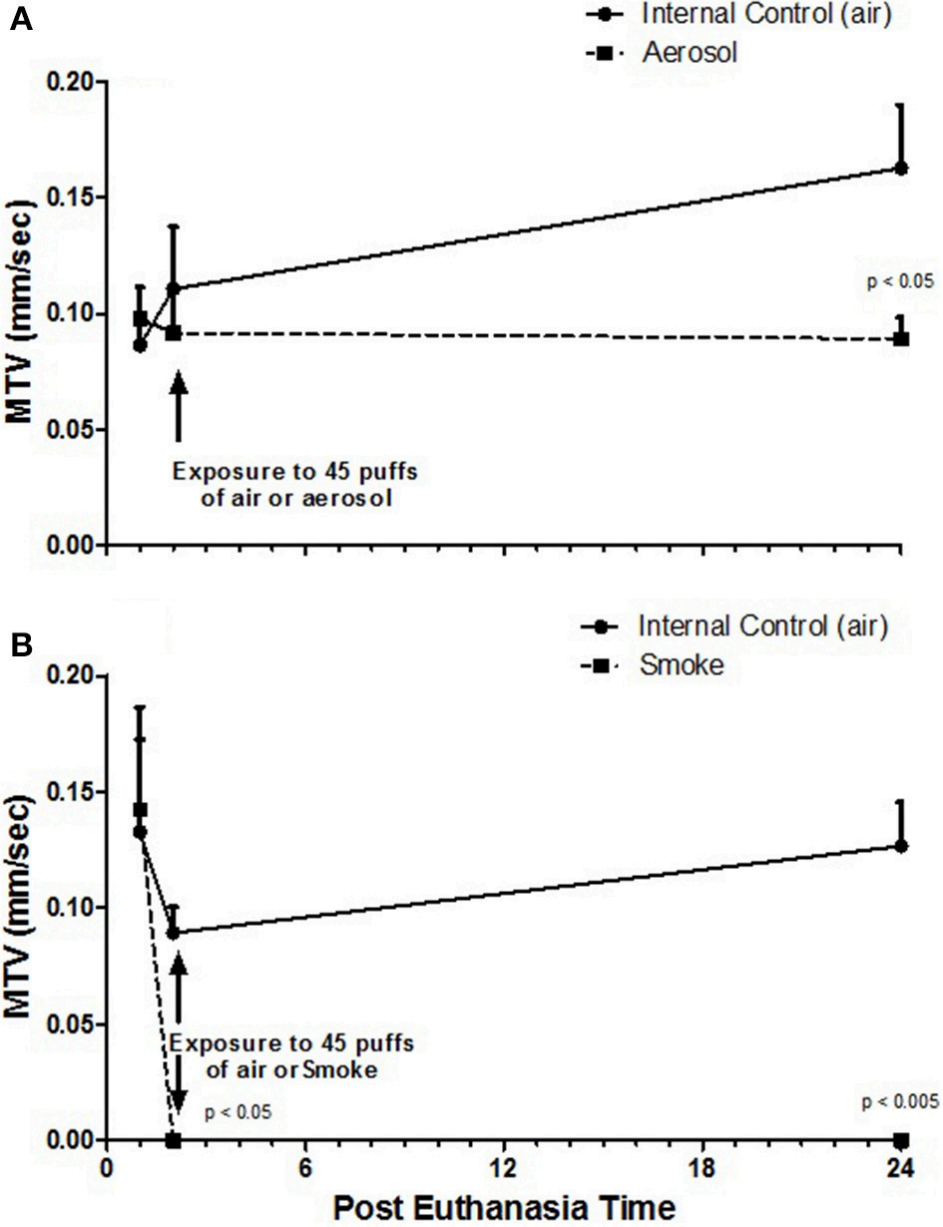
MTV **(A)** from half-palates exposed to aerosol compared to half-palates exposed to internal control (air) and **(B)** from half-palates exposed to smoke compared to half-palates exposed to internal control (air). MTV values given as Mean ± SE. The given *p*-values indicate statistical significance from internal control.

Table 2 presents MTVs for external control half-palates (i.e., never exposed) and internal control half-palates (i.e., exposed to air) for aerosol and smoke groups at 1, 2, and 24-h post euthanasia. No statistical differences in MTVs exist between any of these control groups, at any of the time points. Figure 2A indicates that MTVs for half-palates exposed to 45 puffs of aerosol is not different from their respective internal controls immediately after exposure, but are significantly lower 24-h post euthanasia (*p* < 0.05). In contrast, MTVs for half-palates exposed to smoke are significantly lower from their respective internal controls immediately after exposure (*p* < 0.05) and 24-h post euthanasia (*p* < 0.005), as shown in Figure 2B.

**Table 2:** Control MTV values.

**Post Euthanasia Time**	**External Control (never exposed)**	**Internal Control for Aerosol**	**Internal Control for Smoke**
1-h (pre-exposure)	0.12 ± 0.01 (*n* = 8)	0.09 ± 0.01 (*n* = 10)	0.13 ± 0.05 (*n* = 8)
2-h (exposure to air)	0.06 ± 0.01 (*n* = 8)	0.11 ± 0.03 (*n* = 9)	0.09 ± 0.01 (*n* = 9)
24-h (post-exposure)	0.09 ± 0.01 (*n* = 8)	0.16 ± 0.03 (*n* = 10)	0.13 ± 0.02 (*n* = 10)

*Values given as Mean ± SE, n, number of half-palates*.

### SEM analysis

**Figure 3:**
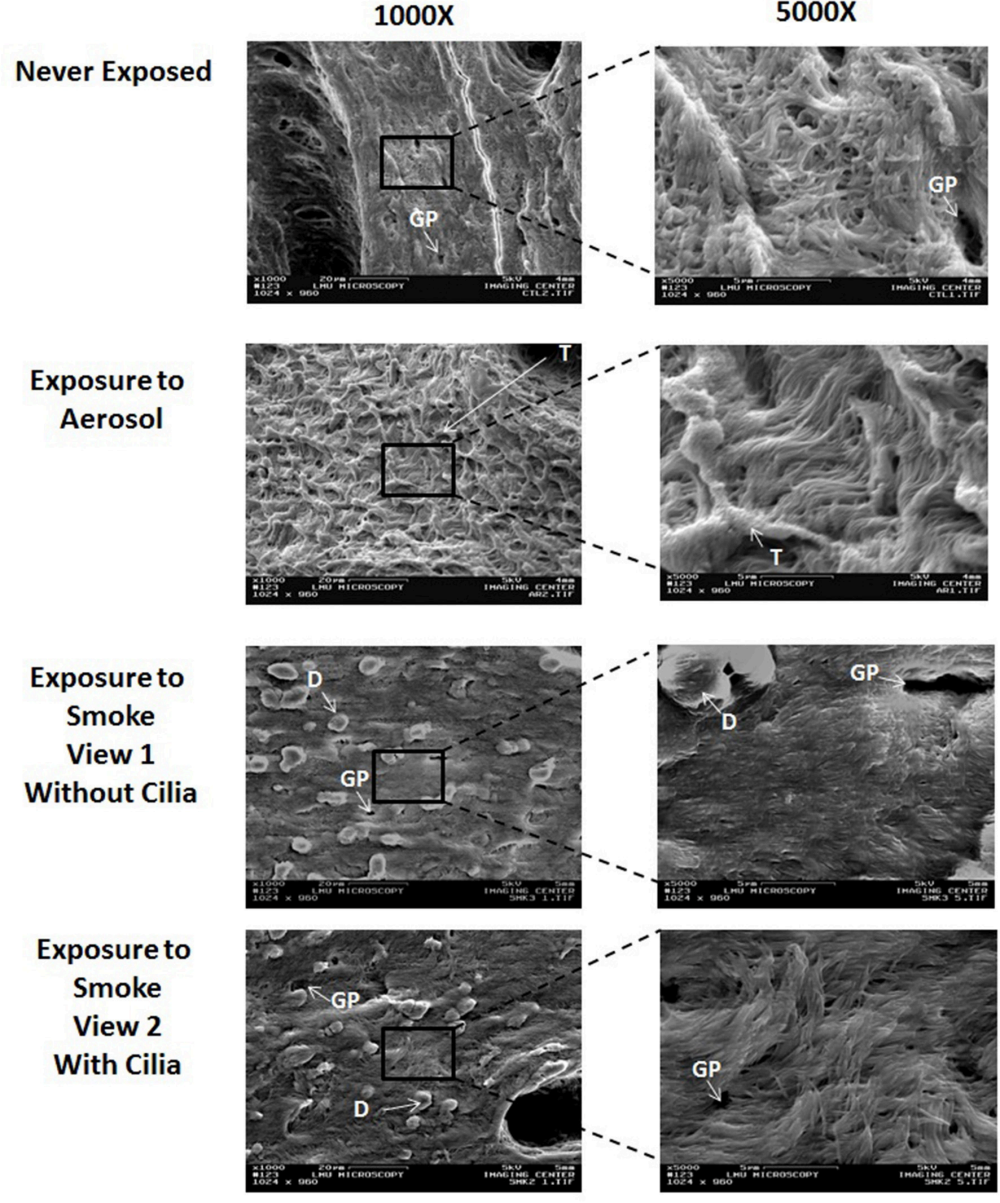
Surface SEM images of never exposed palates compared to palates exposed to 45 puffs of aerosol or smoke at magnifications of 1000X and 5000X. GP, glandular pit; T, Trabecular-like matter; and D, debris.

Figure 3 shows representative SEM images of external control (never exposed) and aerosol and smoke exposed frog palate surfaces at 1000X and 5000X. The never exposed palate at 1000X displays an epithelial surface with many visible glandular pits. At 5000X, the cilia are clearly visible and appear to point upward and away from the epithelial surface. The appearance of the aerosol-exposed palate at 1000X is vastly different, showing a palate caked with matter that is most likely the deposition of puffed aerosol. This matter has a trabecular-like appearance, which obscures the glandular pits. At 5000X, the cilia are visible, but because of the aerosol precipitation, they appear longer and lie flatter to the epithelial surface. While the glandular pits of smoke-exposed palates are still present at 1000X, they are somewhat obscured by smoke-induced debris present on the surface of the epithelium. While cilia are still visible in some areas of smoke-exposed palates, they are conspicuously absent in other areas. At 5000X, the smoke-exposed palate of view 1 is devoid of cilia, consequently revealing a well-exposed keratinized epithelial surface. In contrast, the smoke-exposed palate of view 2 exhibits well defined cilia. In other images (not shown) of smoke-exposed palates, cilia are present but reduced in number.

**Figure 4:**
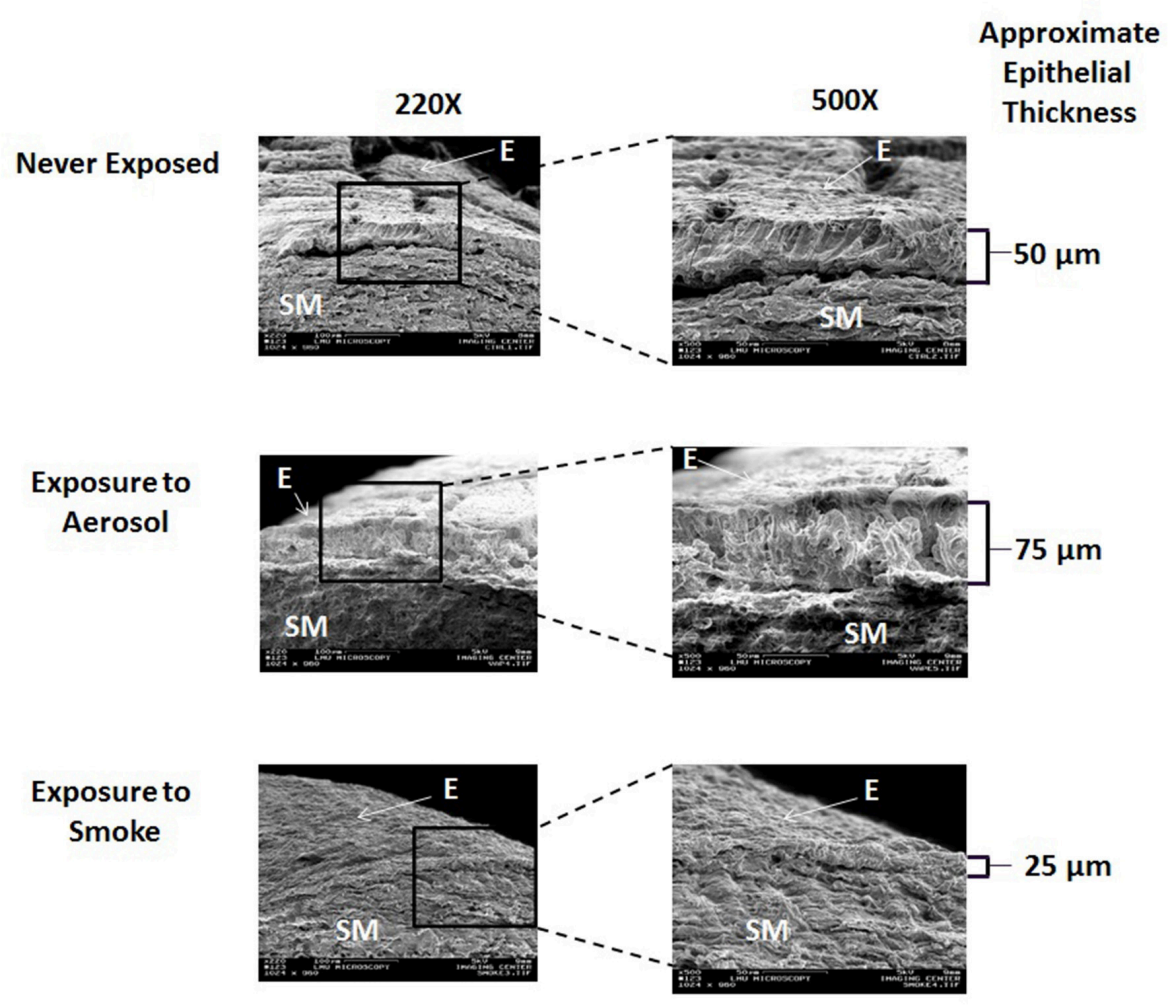
Cross sectional SEM images of never exposed palates compared to palates exposed to 45 puffs of aerosol or smoke at magnifications of 220X and 500X. E, Epithelium; SM, Submucosa.

Representative cross sectional SEM images of external control (never exposed), aerosol, and smoke-exposed frog palates at 220X and 500X are shown in Figure 4. On visual inspection, neither ECIG-generated aerosol nor conventional cigarette smoke appear to affect the integrity of the submucosal architecture as evidenced by the typical arrangement of collagen. However, the epithelium of aerosol-exposed palates appears thicker than the never exposed and smoke-exposed palates due to the addition of aerosol deposition.

**Figure 5:**
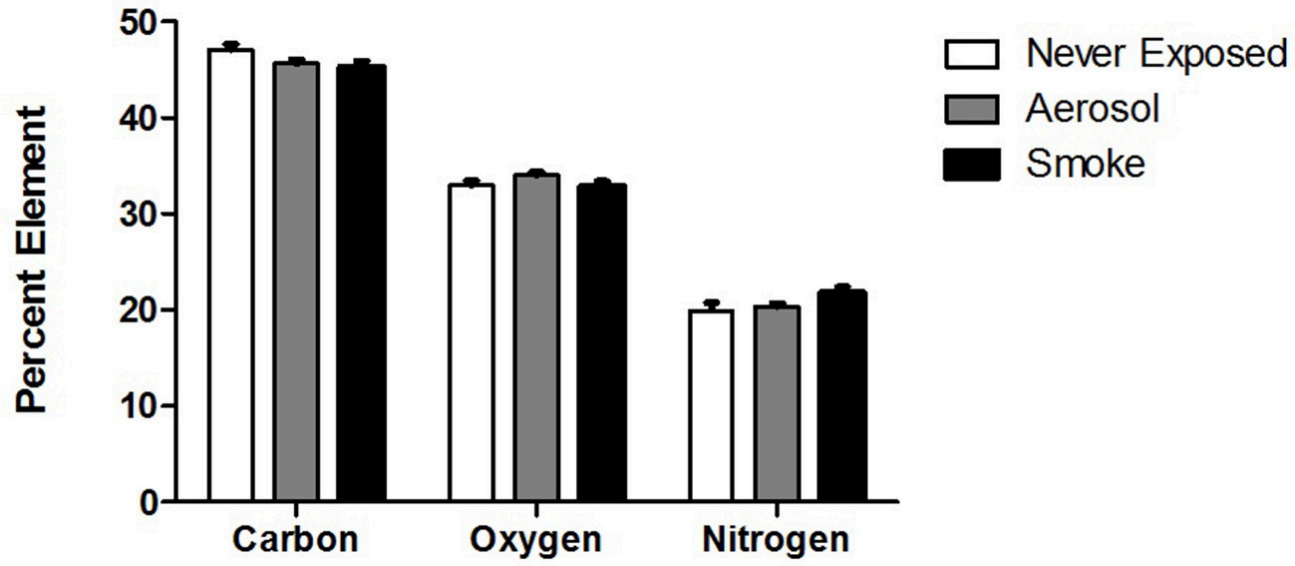
Relative percent surface composition of C, O, and N of never exposed palates compared to palates exposed to aerosol and smoke. Values given as Mean ± SE.

The relative epithelial percent composition of C, O, and N for never exposed palates and palates exposed to ECIG-generated aerosol and conventional cigarette smoke are shown in Figure 5. This data indicates no statistical difference between groups for all three elements. Percent C ranged from 44.1 to 45.4%, percent O ranged from 34.8 to 35.4% and percent N ranged from 19.3 to 20.5%.

## Discussion

This investigation demonstrates that smoke from conventional cigarette smoke inhibits mucociliary clearance of frog palate more dramatically than does ECIG-generated aerosol. Furthermore, SEM analysis of the palates support this finding.

From this study, it is determined that ECIG-generated aerosol has a small dampening effect on the mucociliary clearance of frog palates. Several recent studies support this finding to varying degrees. Using mice in an *in vivo* investigation, Laube et al. (2017) determined that chronic exposure, but not acute exposure, to ECIG-generated aerosol in the presence of nicotine slowed mucociliary clearance. Kumral et al. (2016) reported that individuals who use ECIG devices as a means to quit smoking produced a negative impact on sinonasal symptoms and nasal mucociliary clearance as compared to individuals who do not use ECIG devices. Using various indicators of mucocilialary clearance, to include airway surface liquid volume and cystic fibrosis transmembrane regulator (CFTR) function, Grosche et al. (2016), showed that *in vitro* exposure of normal human bronchial epithelial cells to ECIG-generated aerosol causes mucociliary dysfunction, which is augmented by the presence of nicotine. At this time, it is unclear why MTVs of half-palates exposed to aerosol are lower than their matched internal controls 24-h post exposure, but not immediately after exposure. It is possible that a recovery effect from the stress of palate excision contributes to higher MTVs exhibited by the internal controls 24-h following exposure to air while recovery is masked in palates exposed to aerosol because of aerosol sedimentation on the surface of the palate. The data concerning MTVs of smoke-exposed half-palates indicates a complete shutdown of mucociliary clearance immediately after exposure to smoke. Furthermore, the cilia appear not to recover 24-h later, indicating permanent damage of the ciliated palate. Similarly, Zayas et al. (2004), found close to 100% reduction in MTV immediately after exposure to side-stream smoke from 4 cigarettes and a 100% reduction of MTV 24-h later. Several other reports (Lourenco et al., 1971; Agius et al., 1997; Hessel et al., 2014) also support our study. Hessel et al. (2014) measured the length of cilia from large and small airways and demonstrated that healthy smokers have significantly shorter cilia (7.4 μm) than healthy nonsmokers (7.8 μm) and smokers with chronic obstructive pulmonary disease have even shorter cilia (6.2 μm) than do healthy smokers. From a population of British individuals, Agius et al. (1997) found the mean nasal cilia beat frequency of smoke-exposed individuals (10.6 Hz) to be less than for non-smoke-exposed individuals (11.8 Hz). Lourenco et al. (1971) determined the retention of inhaled ^198^Au-labeled lead particles (≈2 μm) by the trachea/bronchi over a 24-h period and reported that after 1 h of inhalation, the smokers cleared 2.6% of the initial load of particles as compared to 18.5% for non-smokers. These studies are all suggestive of smoke-induced inhibition of mucociliary clearance. From Figure 3, smoke-exposed palates display a mucosal architecture that differs from the never-exposed palates. The smoke appears to disrupt the mucosal surface of the palates and litters it with debris, which is most likely the remnants of exfoliated epithelial cells and not fallout from cigarette smoke. Mixed cellulose ester (MCE) membranes exposed to 45 puffs of cigarette smoke show a C:O:N ratio that differs drastically from unexposed MCE membranes (Palazzolo et al., 2017). However, focused EDX analysis of the debris (data not shown) reveal a C:O:N ratio that is similar to control palates, thus excluding the debris as smoke fallout. While some areas of smoke-exposed palates are completely devoid of cilia, other areas on the same palate exhibit well defined cilia, albeit reduced in size and/or number. This agrees with Zayas et al. (2004), who report a 51 ± 14% loss of cilia from bullfrog palates exposed to four cigarettes, while less than 2% loss of cilia is noted for the control palates.

MTV depends on both coordinated ciliary movement and the physical and chemical nature of the mucous itself. Smoke-induced alterations in the normal function of cilia or changes in the physical and chemical nature of the mucous, or both could lead to deficits in mucociliary clearance. According to Zayas et al. (2004), an increase in the presence of smoke-induced matrix metaloproteinases (MMP) in the mucous may be partially responsible for the loss of mucociliary function and the epithelial disruption of palates exposed to smoke via direct cell-to-cell or cell-to basement membrane connections. MMPs are zinc(Zn)-dependent endopeptidases, known to degrade all types of extracellular matrix proteins. MMP-9, in particular, is associated with a number of pathophysiological processes such as inflammation and fibrosis associated with wound healing and proliferation and is a specific type IV collagenase (Yabluchanskiy et al., 2013). Zayas et al. (2004), found increased activity levels of MMP-9 in the mucous of frog palates exposed to smoke. Similarly, De et al. (2011), reported higher concentrations and activities of MMP-9 in the nasal secretions of children exposed to passive smoke and Chaudhuri et al. (2013) showed the level of sputum MMP-9 to directly correlate with the degree of smoke-induced emphysema. From this evidence, along with evidence showing dietary supplementation of Zn promoting MMP-9 and MMP-2 activities (in the brains of a transgenic mouse model for Alzheimer's Disease; Corona et al., 2010) and Zn-chelation inhibiting MMP-2 activity (in cultured human endothelial cells harvested from the veins of umbilical cords; Huang et al., 2011), it is logical to speculate that the high levels of Zn present in cigarette smoke (Palazzolo et al., 2017) could induce MMPs to disrupt the mucosal surface of the frog palate. High levels of Zn and other trace metals are also known to upregulate the production of metallothioneins (MTs) as a protective mechanism (Klaassen et al., 1999). Evidence also exists showing overexpression of MT increases expression of MMP-9 in a human breast cancer cell line (Kim et al., 2011) and increased presence of Zn(II) on MT increases MMP-9's ability to breakdown collagen (Zitka et al., 2011). Furthermore, our laboratory has recently determined that smoke, but not aerosol, upregulates *mtl-1* and *mtl-2* expression in exposed *C. elegans* (unpublished data).

The cross-sectional SEM images, depicted in Figure 4, demonstrates that the integrity of the submucosal arrangement of collagen in both aerosol-exposed and smoked–exposed bullfrog palates appears to remain intact. This indicates that neither aerosol nor smoke penetrate the mucosal layer of the palate deep enough to have a conspicuous histological effect on the underlying submucosa. From the work of Zayas et al. (2004) and others (De et al., 2011; Chaudhuri et al., 2013; Yabluchanskiy et al., 2013) it appears that immediate epithelial disruption of the smoke-exposed palates is a mucosal phenomenon. However, since the palates were fixed with 2.5% glutaraldehyde in PBS within 24-h of smoke and aerosol exposure, possible long-term effects of submucosal architecture cannot be ruled out.

From this investigation, it is not possible to discern a difference between smoke-exposed and aerosol-exposed epithelial thickness, *per se*, but the trend portrayed in Figure 4 is that smoke-exposed palates have thinner epithelial linings than aerosol-exposed palates. This trend is most likely affected by smoke disrupting the epithelium and aerosol deposition adding to the thickness of the epithelium, respectively. However, this finding is qualitative and subjective, based on visual observation of the SEM images and assumes that the surface of the frog palate is uniform in thickness. It is unfortunate that accurate quantitative measurements of epithelial thickness could not be obtained from the cross-sectional images. Since palate cross sections were prepared using fine scissors, it is impossible to guarantee smooth surfaces and perfect 90° angles required to obtain accurate measurements of thickness.

Evidence supporting deposition of aerosol directly on the mucosal epithelium is shown in a recent publication by Pichelstorfer et al. (2016), who used complex mathematical modeling to explain aerosol and smoke dynamics. Using this model, they demonstrate larger aerosol droplets and more lung deposition associated with ECIG-generated aerosol than for conventional cigarette smoke. They reason that ECIG-generated aerosol has a higher hygroscopic growth rate than does conventional cigarette smoke, thus accounting for the increased droplet size and increased lung deposition. Aerosolized propylene glycol, the main component of the E-liquid used in the present investigation, is hygroscopic (Niven et al., 2011). Analysis of aerosol and smoke dynamics within exposure chambers (as used in our study) is simple by comparison to analysis of aerosol and smoke dynamics within intact respiratory airways. Nevertheless, the hygroscopic nature of aerosolized propylene glycol, whether *in vivo* or *in vitro*, would allow for greater precipitation of ECIG-generated aerosol than conventional cigarette smoke (Pichelstorfer et al., 2016) and could explain the increased deposition observed in the aerosol-exposed palates.

While aerosol precipitation on the mucosal surface contributes to epithelial thickening, it may not be the only means by which epithelial thickness increases. Suber et al. (1989) explain that propylene glycol thickens the respiratory epithelium by increasing the number of goblet cells or increasing the content of mucin within the goblet cells. These findings, observed on autopsy of Sprague-Dawley rats at the end of 90 days exposure (6h/day, 5 days/week) to propylene glycol, are unlikely to be responsible for the findings of the present study since our frog palates were exposed to a single regimen of 45 puffs of aerosol, which would not allow time for the proliferation of goblet cells. Additionally, Suber et al. (1989) reported nasal hemorrhaging to which they attribute to the subchronic nose-only inhalation of propylene glycol. They speculate that dehydration, brought about by long-term exposure to propylene glycol, is responsible for hemorrhaging of the nasal cavity along with subsequent histological changes. Dehydration of the respiratory airways due to the hygroscopic nature of aerosolized propylene glycol, could also explain the compensatory salivation observed in Beagle dogs exposed to long-term inhalation of aerosolized propylene glycol (Niven et al., 2011). On the other hand, Fain et al. (2015) demonstrate that exposure of cultured Calu-3 airway epithelial cells to both aerosolized and unaerosolized vegetable glycerin (another major component of E-liquid) inhibit CFTR-dependent ion transport. It is likely that the presence of nicotine (Grosche et al., 2016) or specific flavorings (Sherwood and Boitano, 2016) in the E-liquid inhibit CFTR function to varying degrees. These finding could also account for dehydration of respiratory airways and the xerostomia, cough and throat irritation reported by many ECIG users (Baweja et al., 2015). These published reports provide further evidence to suggest that deposition of ECIG-generated aerosol adds to the thickness of the respiratory epithelium, which subsequently could affect mucociliary clearance.

The percentages of C, O, and N (shown in Figure 5) in never exposed palates and palates exposed to ECIG-generated aerosol or conventional cigarette smoke are similar, indicating that the deposition of aerosol and smoke onto the frog palates within the chambers is too low to significantly alter the elemental composition of the palate surface. The larger volume of the exposure chambers, compared to the human oral cavity, attenuates the deposition of C, O, and N onto the palate surface. The volume of the human oral cavity; as *per* the height, width, and depth dimensions of a wide-open mouth; (Kaufman and Farahmand, 2006) is approximately 230 cm^3^ and the volume of a mouth positioned for puffing would be even less. The volume of the chambers used in our study is approximately 2,100 cm^3^. Since the volume of the exposure chambers are nearly tenfold greater than the volume of the wide-open mouth, the amount of aerosol or smoke deposition onto the palates is far less than realistically expected in the anatomically intact mouth. When puffing, the mouth is closed, effectively reducing the volume of the oral cavity and consequently increasing the probability of aerosol or smoke deposition. From this over simplistic view, it is conceivable that smoking or vaping would alter the percentage of C, O and N atoms detectable on the surface of healthy human respiratory epithelium, thus amplifying the effects noted in this investigation. According to Pichelstorfer et al. (2016), diffusion is the dominant deposition mechanism for smoke, while inertial impaction and sedimentation are the dominant deposition mechanisms for ECIG-generated aerosol. Translating this information to healthy smokers and ECIG users, the hypothetical implication is that more deposition is likely to occur with ECIG-generated aerosol than with smoke. Consequently, EDX of the palate surface would reveal this as an increase in the total number of C, O, and N atoms, but not their percentages, as previously demonstrated (Palazzolo et al., 2017). On the other hand, burning of tobacco is more likely to alter the C:O:N ratio of the smoke fallout because of oxygen depletion associated with thermal combustion. Thus, EDX of the palate surface would reveal this as a decrease in the percentage of O and an increase in the percentage of C, again, as previously demonstrated (Palazzolo et al., 2017). In our study, cross sectional EDX analysis of exposed collagen was not performed, but given that no differences in the percentage of C, O, and N atoms were detected when surveying a wider field of view associated with the palate surface, there is no reason to suspect differences when canvasing a narrower field of view associated with palate cross sections.

From a physiological perspective, we are confident that cigarette smoke has a more drastic effect on mucociliary clearance, as indexed by MTV, than ECIG-generated aerosol. However, this investigation is not without its limitations. First, MTV values were determined using amphibian and not mammalian tissue. Furthermore, the frog palate is not strictly considered respiratory tissue and because it is an *ex vivo* preparation, does not have an intact salivary flushing mechanism normally present *in situ*. Consequently, the effect ECIG-generated aerosol or smoke have on mucociliary clearance is not exactly comparable to humans. On the other hand, the frog palate has been used for decades as a standard model to analyze mucociliary clearance, because the ciliated epithelium is covered in a blanket of mucus that works in conjunction with cilia very similar to humans (Zayas et al., 2004). The percentages of C (45.4%), O (35.3%), and N (19.3%) of never-exposed palates obtained from our investigation are like the ones published by Maksymowicz et al. (2012) for both human and dog *fascia lata*, indicating that amphibian tissues have a similar C, O and N composition to mammalian species. In humans, they determined the percentages of C, O and N to be 41.3, 33.6, and 18.7%, respectively, and in dogs 44.9, 31.9, and 17.8%, respectively, further contributing to the long-held belief that the frog palate paradigm is a useful model to study mucociliary clearance in humans. Another limitation is that our study utilized only one brand of E-liquid. It is entirely possible that other brands of E-liquids, particularly those brands containing additional flavorings, could have more severe effects on mucociliary clearance. According to Bahl et al. (2012), cytotoxicity of human embryonic stem cells exposed to ECIG refill solutions is primarily due to the number and concentration of chemicals used to flavor the fluids. Leigh et al. (2016) report similar results, indicating that flavorings, especially strawberry, contribute significantly to cytotoxicity of NCI-H292 cell line (derived from a lymph node metastasis of a pulmonary mucoepidermoid carcinoma) induced by ECIG-generated aerosol, albeit to a less degree than cigarette smoke. Sundar et al. (2016) indicate that oral epithelial cells and periodontal fibroblasts elicit inflammatory and prosenescence responses to a greater degree when exposed to ECIG-generated aerosol with flavorings than without. Other minor limitations include the fact that ECIG-generated aerosol and conventional cigarette smoke, by nature, are not identical. Thus, vaporization of E-liquid, compared to combustion of tobacco result in exposure chambers with different physical environments, such as temperature and humidity (Palazzolo et al., 2017) both of which could confound MTV results. Finally, the results of this investigation report only a minor short-term effect of ECIG-generated aerosol on MTV using an *ex-vivo* system. Aerosolized propylene glycol in intact live animals, as reported by Niven et al. (2011) in Beagle dogs and Suber et al. (1989) in Sprague Dawley rats, are known to have long-term effects on respiratory epithelium, to include histological alterations, dehydration of airways, and nasal hemorrhaging, all of which could affect mucociliary clearance more drastically over time.

In conclusion, our MTV results indicate that cigarette smoke affects mucociliary clearance of the frog palate more severely than ECIG-generated aerosol. From an acute physiological perspective, ECIG-generated aerosol inhibits mucociliary clearance modestly, as illustrated by MTV persistence immediately and 24-h after exposure, while conventional cigarette smoke completely shuts down mucociliary clearance immediately after exposure with no evidence of recovery 24-h later. In general, SEM images support these acute MTV findings, especially regarding smoke-induced epithelial disruption. The SEM images of palates exposed to ECIG-generated aerosol suggest that chronic exposure of aerosolized E-liquid could potentially have more deleterious and lasting effects on mucociliary clearance. Although further investigations are required to confirm our *ex vivo* studies, the existing evidence is quite telling, considering the magnitude of morphological changes observed over the 24-h/45 puff experiment. Accepting that there is no circulatory supply of defensive elements or nutrient replenishment, the amount of ciliary and epithelial necrosis observed after exposure to smoke over the experimental interval is quite alarming. Perhaps the observed impact of smoking is much more dramatic *ex-vivo* than *in-vivo* due to the absence of protective systemic defense mechanisms. Although it is evident that ECIG-generated aerosol is deposited on the mucosal surface of the frog palates, there is no evidence to suggest underlying epithelial damage.

## Author contributions

DP: Devised the puffing protocol and developed the experimental design, had primary oversight of all experiments and wrote the manuscript with the editorial assistance of the other authors. JN: Performed elemental analysis of frog palates. EE, AC, and JD: Collected the MTV data. SK: Captured and analyzed SEM images.

### Conflict of interest statement

The authors declare that the research was conducted in the absence of any commercial or financial relationships that could be construed as a potential conflict of interest.

## Footnotes

^1^AVMA Guidelines for the Euthanasia of Animals: 2013. Edition. Available online at: https://www.avma.org/KB/Policies/Documents/euthanasia.pdf.
